# Ear Vertigo in the Horse, Induced by Aspergillus Nigricans

**Published:** 1892-04

**Authors:** Thos. B. Goodall

**Affiliations:** Christchurch, Hants


### EAR VERTIGO IN THE HORSE INDUCED BY ASPERGILLUS NIGRICANS.

By Thos. B. Goodall, F. R. C. V. S., Christchurch, Hants.

When a new theory is propounded, it is the privilege of the veterinary surgeon to search for himself and "see whether these things are so." Personally it is my habit, and, I believe, a right one, to take the first opportunity of testing the trustworthiness of the theory, and of doing the little that lies in my power either to refute it, or to throw light on what may have been left in partial obscurity. It is only by such means that truth is unrolled and revealed to generation after generation of mankind.

It will be understood, therefore, what my feelings and motives were when the first case of Ear Vertigo came under my notice, after reading Dr. Fleming's article, and the subsequent correspondence on the subject in the *Veterinary Journal.*

I should have deemed it much more satisfactory if, as I suggested early in the history of the case, the subject could have been purchased and placed under the supervision of some noted pathologist, as the testimony of an obscure country practitioner might not be credited.

My case is, in most points, a very ordinary one; all practitioners must have seen dozens of cases of what I was taught, and have always looked upon and designated "canker" of the ear.

It may at once be recognized by the horse holding the ear back, giving evidence of the presence of some irritant, and the animal is always averse to having the ear handled. Stablemen call such horses "touchy" about the ears. On closer examination there will be found a number of bare white patches, or excrescences, of various sizes on the skin, lining the inner aspect of the concha; or, in horses whose ears have been neglected, these organs will be found to have in them large quantities of desquamated epidermic scales, and this condition sometimes extends into the external auditory canal.

Hitherto I have looked upon these cases as practically incurable, and have advised as little pulling about of the ears as possible. In bad cases I have had them kept clean, and have applied anodyne lotions for the purpose of alleviating the irritability, though I have never expected recovery. I have known cases of spontaneous recovery, but after reading Dr. Fleming's article in *The Journal*, I was particularly interested with the case I am now about to describe.

On December 12th last I was asked to visit a pony. I was told that she had fallen over when having the harness put on. I found my patient—a seven-year-old forest mare—in low condition. She had been turned out to grass during the day and taken in at night, when she recived a little hay. She was used occasionally for light agricultural work. On this particular day, while the harness was being put on, the pony was reported to have tumbled over, but to have quickly regained her feet; as she *nearly fell* again while being put in the cart, it was deemed advisable to take her out of harness and send for me.

The appearance of the pony was striking and remarkable. The head was constantly inclined to the right side, and it was only with difficulty that it could be brought round in the opposite direction. The right ear was kept lying back, and every now and then

(about every half-hour) the pony would *almost* tumble over in a fit; especially would she do this when excited, although she never quite "lost her feet."

She was standing in a dark stall, and I went up to her on the near tide to take pulse, etc., and was endeavoring to bring the head round towards the near side, to note color of mucous membranes, etc., when one of the epileptiform fits was at once induced. She suddenly fell away towards the off side, and backwards, and after a violent struggle, lasting probably a few seconds, but which appeared a much longer period of time to me in my uncomfortable position, *she nearly fell, but not quite.* She quickly regained the erect position, and was quiet again almost directly. In this and subsequent visits I could induce these "fits" as often as I liked, by bringing the head round towards the near side.

The skin on the inner aspect of the conchæ of the ears was covered by what at first sight had the appearance of warty growths, a condition usually designated "canker." I was given to understand that there had always been a difficulty in handling the ears. The pony's general health was good, though there seemed at times to be a little "reeling" of the hind-quarters while walking or turning.

I had seen dozens of these "ear" cases before, but had never known one to induce anything approaching "staggers;" but with my mind dwelling on Dr. Fleming's paper, and Mr. Sewell's article on the "Symbiots in the Ear of the Dog," I felt that this was an opportunity to ascertain whether this affection might not be parasitic. With some difficulty the head was secured, and after making a careful examination of the ear with my lens, I took some scrapings of the epidermis from under the exfoliated scales. I was quite prepared to find Acarians, but in this I was disappointed.

On every one of the slides on which I had mounted portions of these scrapings, there were found numbers of dark-colored, round bodies, among and on the epidermic scales; they were also noticeable on the corteces of such hairs as were placed on the slides. I was quite satisfied that these were the fructifying parts of a fungus, but, as I could detect neither mycelium nor hyphæ, I hesitated to publish the case until the identity of the fungus could be more clearly established.

Having now made a most thorough investigation, I have no hesitation in pronouncing it to be the endogenous variety of the Aspergillus.

Although there is no record of these having been recognized as a cause of disease in our animals, it has been adduced as a cause of acute Otitis in man.

That I am correct in my diagnosis of the nature of the fungus, is abundantly proved by the case responding so readily to treatment.

The ears were cleansed occasionally, and *Sulphur* was freely applied in the form of ointment to the conchæ, and the dry powder to the auditory canal; and in a fortnight I had the case under control, and the pony cured, which, after all, is the point of most practical importance.

Since that time there has been no recurrence of the "fits;" the pony can be brought round to the "near" as readily as to the "off" side; the ears are carried naturally, and the hair has grown.

Mode of procedure in the identification of the fungus:—

The first step was to get fresh, clean scrapings from the ears.

Some of this was mounted *dry*, and in some specimens (and I mounted dozens altogether), only a *few* of the black bodies were visible; but in looking at these same dry specimens in three days' time, the numbers of the fungi had increased very considerably, proving them to be independent living organisms.

Then some scrapings were treated by immersion in Liq. Potassæ, and the fungi were brought out more clearly as prominent black bodies, varying in size, the largest being about $\frac{1}{4}$ and the smallest $\frac{1}{32}$, the size of an epidermic scale.

The next step was to obtain a scraping of "Favus" from a calf, and mount slides dry, and treat with Liq. Potassæ, for comparison; but there was no point of agreement between these and the "ear fungus."

I next mounted specimens of different *moulds*, the *Mucor penicillium*, &c., and in the Aspergillus I found what I was looking for; for amongst the beautiful arrangement of spores, hyphæ, and mycelium, some of these same black bodies were very noticeable—not by any means in all the specimens, but in two or three of them. Here, then, was the clue.

Further specimens from the ear were now mounted, and just on one of the slides I find spores and a faint net-work of mycelium.

The records of the life-history of the fungus are now searched, and we find it is reported to be highly polymorphic and that it sometimes bears endogenous, and at others exogenous spores. This agrees exactly with the results of my own observations in the present case.

The search is carried a little further, and we find it has been recorded that the *Aspergillus nigricans* has been found in the human ear, and that it has been the cause of acute Otitis.

We could not call this a case of Menier's disease, for we must know that it would not be possible for this fungus to gain an access to the regions of the ear on the inner side of the Tympanum; but its presence, in this instance, induced exactly such a train of symptoms as described in the article in the *Journal.* It may have been from sympathy or from some other cause, but having detected a fungus, my business was to get rid of it; and knowing the specific action of Sulphur in destroying this particular form of organism, I was induced to give it a trial, and, as will be observed, with most satisfactory results.

In pursuing these investigations, it has often occurred to me what a wealth of real pleasure and solid improvement is missed by the many who take everything for granted, without themselves searching into some of these most beautiful and charming, though hidden things of Nature.

I ought to mention that the bulbs of the hairs were always found diseased, having the appearance of a bundle of faggots tied together, with the ends irregularly projecting.—*From The Veterinary Journal.*

